# Factors influencing the retention of participants in online cancer screening training programs in India

**DOI:** 10.1186/s12909-020-02144-y

**Published:** 2020-07-13

**Authors:** Kavitha Dhanasekaran, Roshani Babu, Vipin Kumar, Shalini Singh, Roopa Hariprasad

**Affiliations:** 1 Department of Clinical Oncology, ICMR- National Institute of Cancer Prevention and Research, I-7, Sector 39, Noida, Uttar Pradesh 201301 India; 2 ICMR- National Institute of Cancer Prevention and Research, Noida, Uttar Pradesh India

**Keywords:** ECHO-online cancer screening course, Retention in online course, Attrition of participants in online course, Cancer screening course

## Abstract

**Background:**

Online courses have broken the boundaries in imparting knowledge. While in western countries e-learning in medical education is well accepted, it is still an upcoming field in low- and middle-income countries like India. Attrition is a major threat to online courses world-wide. The objective of this article is to share the experiences in conducting online cancer screening courses, reasons for attrition and ways to improve retention.

**Methods:**

Online training program in preventive oncology for medical professionals is being conducted since 2017, which is 14-week long with weekly one-hour sessions and specific curriculum for healthcare professionals. Since the retention of participants was a major challenge in all the courses, a short online survey was conducted to identify the reason behind quitting the course and suggestions to improve retention. The data was analyzed in November 2019.

**Results:**

Among 614 enrolments, 26% (159/614) refused to attend the course and only 55% (252/455) completed the course successfully. Among the attendees 52% (238/455) were females, 71% (325/455) were from the public sector and 71% (324/455) were non-specialists. The attrition was high among non-specialists 49% (160/324), male participants 57% (124/217) and public sector doctors 52% (170/325). The main reasons for quitting the course were high workload in the health facilities 75% (52/69) and poor internet connectivity 12% (8/69).

**Conclusion:**

The internet connectivity should be strengthened in all the healthcare centers to facilitate e-learning. A dedicated time-slot should be allotted to the providers for e-learning and updating their knowledge in addition to their routine work.

## Background

The cyberspace and learning opportunities through online courses are among the most amazing things that have happened in academics in the twenty-first century. Online courses have broken the boundaries of culture, distance, time and money in imparting knowledge. This revolution in education has aided many students and young professionals to overcome the barriers of conventional classroom learning [1]. The blooming insurgence of sharing knowledge has been the attraction among curious learners and has drastically increased the enrollment to online courses [2, 3].

In conducting online courses technology is used diversely. In courses by Massive Open Online Courses, pre-recorded lectures are utilized for teaching [4, 5]. This particular technique is a privilege for part-time learners as it enables self-paced learning. On the contrary, online courses conducted using the telementoring platform like Extension for Community Healthcare Outcomes (ECHO) run live sessions using the online space [6–8]. This non-conventional approach which provides the luxury of a classroom learning experience in online courses is a boon to the learner community. The success of online courses may depend on various factors but the attrition of the participants is a universal threat for such courses [9–11]. In western countries, e-learning in medical education had been adopted decades ago and it continues to be well accepted even now [5, 12, 13]. Considerable literature, with rich information on the demographics, trends, and behaviors of the participants in terms of retention, merits, and demerits of online courses are available. However, data on online courses conducted in the Indian context exclusively in the field of preventive oncology are limited. The objective of this article is to share the experiences in conducting such courses in India, and the practical challenges faced and addressed in enabling or aiding participants to complete the course.

## Methods

The National Institute of Cancer Prevention and Research (ICMR-NICPR) conducts blended training in preventive oncology for medical professionals since 2017 which comprises online and in-person training. The online course uses free downloadable Zoom software, a knowledge transfer tool and the ECHO model teaching platform in the course wherein the attendees referred to as “spokes” and the team leading the course as “hub”. This “Hub and Spoke” model follows “All Teach All Learn” policy, encouraging discussions without hierarchy [14, 15]. Each course is 14-weeks long with a 60-min live session once a week conducted in the English language. A typical session starts with didactics by the subject expert, followed by case presentation by participants and ends with discussions. The spokes attend the sessions with a specific meeting identification number. The basic course has three modules that deal with cervical, breast, and oral cancer screening. The advanced course for gynecologists deals with cervical and breast modules, while the dentists’ course is on oral cancer screening and tobacco cessation. On successful completion of the online course, the participants attend a three-day face-to-face workshop for skill building. After completing this comprehensive training, the attendees are empowered with essential knowledge and competence to perform cancer screening. Participants receive two certificates one after completion of online courses and the other after completing hands-on training.

The course was open for all interested candidates in 2017. Later the course evolved with specific modules for targeted audiences such as medical officers, gynecologists, and dentists. In 2018, training providers from the public sector began with nominations from the National Health System Resource Center. Recognizing the potential of this program, the Ministry of Health and Family Welfare (MoHFW), Government of India designated ICMR-NICPR as a nodal center for the cancer screening training program in the year 2019. The MoHFW sent a communiqué to all States to nominate Medical Officers (MO) serving in public sector for this course.

The systematic training of MOs started with the states of Chhattisgarh, Bihar, Goa, and Tripura. The State Program Officers of Non-Communicable Disease Cell provided the nomination to the hub team. The hub team communicated information about the course and their nominations to the participants. Currently, MOs who are nominated by their respective States are trained in cancer screening using the hybrid model. The details of the courses are enclosed as a Supplementary document.

To assess the outcome of the training program from 2017 to 2019, and to know the reason for high attrition rate in the program, we devised a quantitative questionnaire with three questions for a short online survey to understand (a) the reasons for quitting, (b) suggestions to improve retention and (c) the district and state where they were employed (enclosed as a Supplementary document). We piloted the questionnaire among a few participants and incorporated their suggestions. The final questionnaire was reviewed and approved by senior researchers and the course director. The survey was conducted using Survey Monkey software and was sent to all participants who did not complete the course. The time-line to respond to the survey was 1 month considering their busy schedule. The two-year data, including all the courses was analyzed in November 2019.

### Statistical analysis

For the ease of analysis, the cohorts were categorized into three groups:

Group 1: 2 cohorts of gynecologists (Specialists-advanced group), Group 2: 1 cohort of dentists (Specialists - advanced group), and Group 3: 8 cohorts of medical officers (Non-specialists- Basic group).

The data was analyzed using SPSS version 19.0. The categorical variables are analyzed descriptively, and the results presented as percentages. A Chi-square test was used to analyze quantitative data and *p*-value ≤0.05 was considered to be statistically significant. Cramer’s V test was applied to find out the strength of association (very strong association: > 0.25; strong association: > 0.15; moderate association: > 0.10; and weak association: > 0.05).

## Results

Among 614 participants who were either nominated by the state authorities or voluntarily enrolled in the courses, one-fourth (159/614 [26%]) refused participation in the course (refused). The refusal was maximum (39%) in group 3 and minimum (17%) in group 2 (Table 1).

**Table 1:** Details of enrolled participants in various groups

Status	Group 1		Group 2		Group 3		Total	
	n	%	n	%	n	%	n	%
Enrolled	131	75.3	209	83.3	115	60.8	455	74.1
Refused	43	24.7	42	16.7	74	39.2	159	25.9
Total	**174**	**100.0**	**251**	**100.0**	**189**	**100.0**	**614**	**100.0**

Group 1: 2 cohorts of gynecologists (advanced group)

Group 2: 1 cohort of dentists (advanced group)

Group3: 8 cohorts of medical officers (Non-specialists- Basic group)

**Fig. 1:**
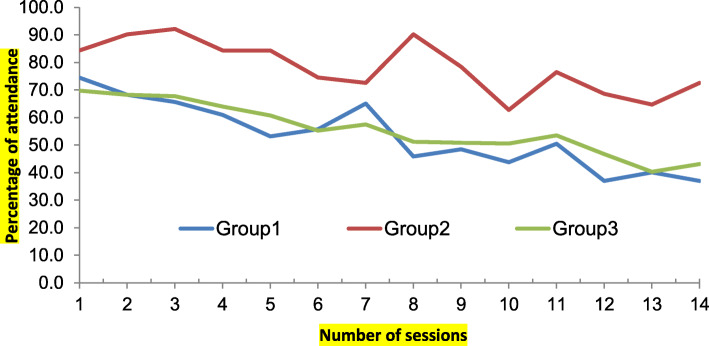
Attrition of participants from all the courses. Footnote: Group 1: 2 cohorts of gynecologists (advanced group). Group 2: 1 cohort of dentists (advanced group). Group 3: 8 cohorts of medical officers (Non-specialists- Basic group)

Among the attendees of the courses, the dropouts were noted as the highest among the group 3 and lowest among group 2. The details are plotted on a graph (Fig. 1). Among the enrolled participants in all 3 groups combined, 52% were females (238/455), 71% (325/455) were from the public sector and 71% (324/455) were non-specialists. Out of all enrolled participants, the completion rate among the female participants was high which was statistically significant 67% (159/238), *p* < 0.001; Cramer’s V: 0.24 (strong association), participants from private sector had higher completion rate than the public sector 75% (97/130), *p* < 0.001; Cramer’s V: 0.24 (strong association), completion rate was higher among the specialists as compared to the non-specialists 67% (88/131), *p* < 0.005; Cramer’s V: 0.15 (strong association),and the completion rate among paid participants were significantly better than the unpaid participants 76% (132/174), *p* < 0.001; Cramer’s V: 0.32 (very strong association), (Table 2).

**Table 2:** Details of course completion rate in various categories

	Completed n (%)	Not Completed n (%)	*p*-value (chi-square)	Cramer’s V (association)
Gender				
Male (*n* = 217)	93 (42.9)	124 (57.1)	*p* < 0.001	0.24 (Strong)
Female (*n* = 238)	159 (66.8)	79 (33.2)		
Sector				
Public (*n* = 325)	155 (47.7)	170 (52.3)	*p* < 0.001	0.24 (Strong)
Private (*n* = 130)	97 (74.6)	33 (25.4)		
Category				
Paid (*n* = 174)	132 (75.9)	42 (24.1)	*p* < 0.001	0.32 (Very Strong)
Unpaid (*n* = 281)	120 (42.7)	161 (57.3)		
Qualification				
Specialist (*n* = 131)	88 (67.2)	43 (32.8)	*p* < 0.005	0.15 (Strong)
Non-specialist (*n* = 324)	164 (50.6)	160 (49.4)		

### Survey results among participants who dropped out of the course

The survey results of the participants who discontinued the course are given in Table 3.Among all the dropouts and refusals to whom the online survey was sent the response rate was 27% (69/258). Major reasons for quitting/refusal among the respondents were high workload 75% (52/69) and poor internet connectivity 12% (8/69). The state of Tripura and Chhattisgarh accounts for 73% (38/52) of refusals due to a high workload. The participants from Tripura reported the maximum internet connectivity issue 100% (8/8).

**Table 3:** State-wise results of the survey among participants who dropped out of the course

	Reasons for quitting (*n* = 69)						Suggestions to improve the retention (*n* = 69)						
State	Busy at work	Not interested	Unable to understand the subject matter	Other	Network Issue	Total	Change of time	Change in the manner the didactic is delivered	Change in the format of the course	Less number of sessions	Module-wise courses for each type of cancers: cervical, breast and oral	Other	Total
Bihar	1	0	0	0	0	1	1	0	0	0	0	0	1
Chandigarh	1	0	0	0	0	1	1	0	0	0	0	0	1
Chhattisgarh	19	0	0	2	0	21	13	0	2	3	0	3	21
Daman & Diu	1	0	0	0	0	1	1	0	0	0	0	0	1
Goa	2	0	0	0	0	3	2	0	1	0	0	0	3
Gujarat	1	0	0	0	0	1	1	0	0	0	0	0	1
Jharkhand	1	1	0	0	0	2	2	0	0	0	0	0	2
Manipur	1	0	0	1	0	2	1	0	0	0	1	0	2
Orissa	1	0	0	0	0	1	1	0	0	0	0	0	1
Sikkim	1	0	0	0	0	1	1	0	0	0	0	0	1
Tamil Nadu	1	0	0	0	0	1	1	0	0	0	0	0	1
Tripura	19	0	1	3	8	30	16	2	2	3	2	5	30
Uttar Pradesh	2	0	0	1	0	3	3	0	0	0	0	0	3
West Bengal	1	0	0	0	0	1	0	0	0	1	0	0	1
Total	**52**	**1**	**1**	**7**	**8**	**69**	**44**	**2**	**5**	**7**	**3**	**8**	**69**

The other reasons for discontinuation of the course 10% (7/69) were illness, family issues, quitting job to join higher studies. Very few indicated “not interested” 1% (1/69) and not being able to understand the subject 1% (1/69). Sixty-four percent (44/69) participants requested for change of time of the course, of which 36% (16/44) were from Tripura and 30% (13/44) from Chhattisgarh. Few others requested for change in the way the course was conducted 7% (5/69) and preferred a shorter course duration 14% (10/69).

## Discussion

Our study revealed some interesting facts as follows: Female participants were found to be more committed towards the course. Earlier studies also concur with our results [16]. Zdravkovic et al. in his recent publication, reports that women show more interest in clinical work compared to their male counterparts [17]. This could be one of the reasons for the better adherence of female participants in our course. Retention was better among the specialists, especially among the dentists. The dental specialists were curious learners and complied with the course completion requirements [18].

Among those unable to continue the course, the majority was from difficult terrain areas with internet issues and increased workload at health facilities. Many expressed their interest to continue, provided the time of the course could be changed to after work hours, preferably in the late evenings. A few requested for a shorter duration of courses.

While conducting these courses, the major obstacle was low retention. Some salient reasons behind the attrition, the strategies attempted to minimize the drop-outs, and those that improved the retention are listed here:

### Constraints

Technical/technological constrains, language barrier, non-availability of devices, poor internet connectivity, increased workload among doctors were few important reasons for poor retention of the participants in our courses. The Zoom software has many advanced functions like chatting, polling, electronic hand-raising, etc. This technical complexity was a major hindrance to active participation. We addressed this challenge by introducing a dedicated orientation session to acquaint the trainees with the software and telephonically guidance. Power-Point presentation and videos on the orientation were shared with the participants through email before commencement of the course, enabling them to become familiar with the software at their own pace. Email was the primary mode of communication. However, many participants preferred phone conversations and WhatsApp chat to emails. For seamless communications on course details, information share through the dedicated WhatsApp group for each cohort [19].

English was the medium of instruction in the courses, yet few preferred discussions in Hindi, which is a widely spoken Indian language. The silent spectators with the language barrier were allowed to speak in Hindi. and encouraged to continue the course. The course adopts simple English for communication and teaching. The State-authorities communicated the official nomination list directly to the hub, and the participants were unaware of their nomination. This communication gap led to lot of refusals. Some trainees agreed for enrollment after appraising the details of the course by the hub [19].

The majority of the dropouts were from the northeastern states of India with difficult terrain where the internet connectivity was of great concern. The interrupted connectivity in these areas was a challenge for the participants to continue the course [19]. Participants living in island states like The Andaman and Nicobar Islands reported a lack of/severe connectivity issue. Many had to travel long distances to the mainland for better connectivity.

Identifying alternative service providers with better bandwidth and attending without video (which would require lower bandwidth) were suggested to enable them to stay connected. Since cancer screening is yet to start at many primary health centers (PHC), participants opted-out due to lack of cases to present. The hub provided the cases to these participants to improve retention and provide the opportunity of case-based learning, which increased participants’ interest, curiosity about the topics, and boosted their active participation in the sessions. Majority of the spokes attended the sessions with smartphones, which made them uncomfortable to prepare case presentations on their smartphones. Hence they refused to present cases or requested the hub for help. Self-motivation is the key to e-learning. Diverse work responsibilities were a strong demotivator among the participants. Many were not very keen to update their knowledge and few were multi-tasking during the sessions and losing focus.

When the participants were aware of the no-video option many logged-in without video, some misused this situation and played truant. Despite repeated requests, many continued to attend without video. Lack of eye contact de-motivated experts and was a hindrance to the hub team when they were tracking the attendance. Once the participants achieve the mandatory criteria for certification: attending minimum of 10 sessions and presenting a case, the retention dropped drastically to as low as single-digits in a few courses [19]. This discouraged the hub and the faculties. However, the endurance of the hub in motivating attendees acted as the stimulus for the course. Various government health schemes in rural India increased the job responsibilities of the providers. Many expressed their unwillingness to attend another new program despite the nominations. Convinced participants on the necessity of the course enrolled for the course, but a significant number were irregular in their attendance. Mostly, the PHCs have a single doctor who caters to a few hundred patients every day. This strenuous routine makes it difficult for them to continue learning. Conflict of dates with any official proceedings also was a reason for them to miss sessions. In such situations, a recording of the particular session was shared on request.

### Factors augmenting the retention of participants

Few State Nodal Officers (SNO) were actively involved in the course. Team leaders taking ownership of the program was incitement and improved the retention of participants from that particular state. In 2017, the course was free of cost. Due to a very high dropout rate, a nominal registration fee of Indian Rupees1000 (approximately 14 US Dollars) was introduced in subsequent cohorts. This improved the retention rate significantly. However, currently, ICMR-NICPR has adopted a free teaching policy for all since the Government of India has designated ICMR-NICPR as the nodal center for training in cancer screening. Female participants were generally regular in their attendance. This was a significant factor for better retention in some courses.

The attrition was substantially high among the nominated providers from the public sector. This situation could improve if the doctors designated for cancer screening at the grass-root level are sensitized about the magnitude of the cancer burden in our country and the need for screening at the primary healthcare level. During the official gatherings, the doctors may be briefed about the guidelines on screening, and management of the preventable cancers, the government’s mission, and vision to minimize the morbidity and mortality of the preventable cancers in our nation, update on roll-out of PBCS, necessity of structured training to gain knowledge and confidence in cancer screening and the importance of the role which the physicians can play towards prevention and early detection of cancer, etc.

The providers should be given the liberty to choose the days to attend the course. These measures will encourage willful learning among the health care professionals and have ownership of the program. The state officials can form a cohort of doctors with a similar choice of days for training. Official communication with the participants about their nomination, informing them of the start date and time of the course along with the curriculum well in advance is crucial. This will enable them to be prepared for the course and be committed. On successful completion, the MOs can be incentivized as a token of appreciation by the state government for the shared commitment towards the cancer-free nation. These initiatives will spur agog learners and act as a catalyst to efficiently train the medical workforce and roll out an effective nationwide cancer screening program.

#### Limitations

The results of this study are mainly elicited using quantitative methods to know the reasons for the high attrition rate in the online cancer screening training program. Ideally this should have been coupled with qualitative component which would have provided the in-depth understanding of the reasons. Due to the unavailability of the doctors who had dropped out of the course and due to the time constraints, the qualitative part could not be incorporated in the study.

## Conclusion

Internet connectivity should be strengthened in all healthcare centers to facilitate e-learning. A dedicated time-slot should be allotted to the providers to attend various online courses and update their knowledge in addition to their routine work. Promoting a continuum of education through online courses among doctors in the public sector will help the government conduct various training programs at their doorsteps, which will be economical for the government and time saving for the providers.

## Supplementary information

**Additional file 1.** Supplementary Document 1: List of courses conducted.

**Additional file 2.** Supplementary document 2: Questionnaire for quantitative study.

## Data Availability

The datasets used and/or analyzed during the current study are available from the corresponding author on reasonable request.

## Abbreviations

ECHO: Extension for Community Healthcare Outcomes
ICMR-NICPR: The National Institute of Cancer Prevention and Research
MoHFW: Ministry of Health and Family Welfare
MO: Medical Officers
IEC: Institutional ethics committee
PHC: primary health centers
SNO: State Nodal Officer

## References

[1] Mckenna L (2012). The big idea that can revolutionize higher education: “MOOC”. Atl Bus.

[2] Allen E, Seaman J. Going the distance: online education in the United States. J Mod Lit. 2011;26(3):11–27. 10.1353/jml.2004.0031..

[3] Gaebel M. MOOCs massive open online courses by Michael Gaebel January 2013. Eur Univ Assoc 2013. https://supporthere.org/sites/default/files/eua_occasional_papers_moocs_4.pdf. Accessed 15 Dec 2019.

[4] Beginners guide to massive open online courses (MOOCs) | Class Central Help Center. https://www.classcentral.com/help/moocs. Accessed 15 Dec 2019.

[5] Hoy MB (2014). MOOCs 101: an introduction to massive open online courses. Med Ref Serv Q.

[6] Hariprasad R, Arora S, Babu R (2018). Retention of knowledge levels of health care providers in cancer screening through telementoring. J Glob Oncol.

[7] Arora S (2019). Project ECHO: democratising knowledge for the elimination of viral hepatitis. Lancet Gastroenterol Hepatol.

[8] Dhanasekaran K, Babu R, Kumar V, Mehrotra R, Hariprasad R. Capacity building of gynecologists in cancer screening through hybrid training approach. J Cancer Educ. 2019. 10.1007/s13187-019-01589-0. 10.1007/s13187-019-01589-0 31359375

[9] Smith BG (2010). E-learning technologies: a comparative study of adult learners enrolled on blended and online campuses engaging in a virtual classroom.

[10] Herbert M (2006). Staying the course: a study in online student satisfaction and retention. Online J Distance Learn Adm.

[11] Heyman E (2010). Overcoming student retention issues in higher education online programs. Online J Distance Learn Adm.

[12] Liyanagunawardena TR, Williams SA (2014). Massive open online courses on health and medicine: review. J Med Internet Res.

[13] Volandes AE, Kennedy WJ, Davis AD, Gillick MR, Paasche-Orlow MK (2013). The new tools: what 21st century education can teach us. Healthcare..

[14] Chaple MJ, Freese TE, Rutkowski BA, Krom L, Kurtz AS, Peck JA (2018). Using ECHO clinics to promote capacity building in clinical supervision. Am J Prev Med.

[15] Arora S, Thornton K, Murata G, Deming P, Kalishman S, Dion D (2011). Outcomes of treatment for hepatitis C virus infection by primary care providers. N Engl J Med.

[16] Price L (2006). Gender differences and similarities in online courses: challenging stereotypical views of women. J Comput Assist Learn.

[17] Zdravkovic M, Osinova D, Brull SJ, Prielipp RC, Simões CM, Berger-Estilita J (2020). Perceptions of gender equity in departmental leadership, research opportunities, and clinical work attitudes: an international survey of 11 781 anaesthesiologists. Br J Anaesth.

[18] Nethan ST, Hariprasad R, Babu R, Kumar V, Sharma S, Mehrotra R. Project ECHO: a potential best-practice tool for training healthcare providers in oral cancer screening and tobacco cessation. J Cancer Educ. 2019. 10.1007/s13187-019-01549-8. 10.1007/s13187-019-01549-8 31124001

[19] Babu R, Dhanasekaran K, Mehrotra R, Hariprasad R. Leveraging technology for nation-wide training of healthcare professionals in cancer screening in India: a methods article. J Cancer Educ. 2020. 10.1007/s13187-020-01720-6. 10.1007/s13187-020-01720-6 32130665

