# Supplementary material for: Factors influencing the retention of participants in online cancer screening training programs in India
Source: BMC Med Educ. 2020 Jul 13;20:220. doi: 10.1186/s12909-020-02144-y (PMC7359595; doi:10.1186/s12909-020-02144-y)
Supplement: Supplementary file 1 — Additional file 1. Supplementary Document 1: List of courses conducted. [file 12909_2020_2144_MOESM1_ESM.docx]

Supplementary Document 1: **List of courses conducted**

| **Course** | **Enrolled**  **N** | **Refused**  **n** | **Total**  **n** | **Duration**  **(wks)** | **Participants** | **Salient features** |
| --- | --- | --- | --- | --- | --- | --- |
| **ACSTP-G** | 32 | 2 | 34 | 14 | Gynecologists (private sector) | - Registration fee - Pre & Post Training evaluation quiz - Post-session quiz - Blended approach |
| **ACSTP-G2** | 48 | 41 | 89 | 14 | Doctors, dentists, MSWs, public health professionals | - Registration fee - Blended approach - Pre & Post training evaluation quiz |
| **ACSTP-O** | 51 | 0 | 51 | 14 | Dentists, doctors(Public sector& Private sector) | - Registration fee only for Private sector - Blended approach - Pre &Post training evaluation quiz - Post-session quiz |
| **BCSTP-2** | 55 | 12 | 67 | 14 | Doctors, dentists (Public sector) | - Free course - Separate orientation session - Pre &Post training evaluation quiz |
| **CSTP-MO** | 50 | 0 | 50 | 14 | Doctors (Public sector) | - Free course - Separate orientation session - Blended approach - Pre & Post training evaluation quiz |
| **CSTP-MO2** | 36 | 26 | 62 | 14 | Gynecologists(Public sector& Private sector) | - Registration fee for private sector only - Separate orientation session - Blended approach - Pre& Post training evaluation quiz - Post session quiz |
| **CSTP-MO3** | 21 | 4 | 25 | 14 | Doctors(Public sector& Private sector) | - Registration fee for Private sector only - Separate orientation session - Blended approach - state program officers attended the course - Pre &post training evaluation quiz |
| **CSTP-MO4** | 47 | 0 | 47 | 14 | Doctors (Private sector) | - Registration fee - Separate orientation session - Blended approach - Pre & Post training evaluation quiz - Pre & Post session quiz |
| **CSTP-MO5** | 29 | 10 | 39 | 14 | Doctors (Public sector) | - Free course - Separate orientation session - Blended approach - SNO attended the course - Pre & Post training evaluation - quiz - Pre & Post session quizzes |
| **CSTP-MO6** | 23 | 17 | 40 | 14 | Doctors (Public sector) | -do- |
| **CSTP-MO7** | 63 | 47 | 110 | 14 | Doctors (Public sector) | -do- |

Foot note:

ACSTP-G: Advanced cancer screening training program (gynecology)

ACSTP-G2: Advanced cancer screening training program (gynecology, 2^nd^ cohort)

ACSTP-O: Advanced cancer screening training program (Oral)

BCSTP-2: Basic Cancer Screening Training Program (2^nd^cohort)

CSTP-MO: Cancer screening training program for medical officers (1^st^ cohort)

CSTP-MO2: Cancer screening training program for medical officers (2^nd^ cohort)

CSTP-MO3: Cancer screening training program for medical officers (3^rd^ cohort)

CSTP-MO4: Cancer screening training program for medical officers (4^th^ cohort)

CSTP-MO5: Cancer screening training program for medical officers (5^th^cohort)

CSTP-MO6: Cancer screening training program for medical officers (6^th^ cohort)

CSTP-MO7: Cancer screening training program for medical officers (7^th^ cohort)
