# Supplementary material for: Factors influencing the retention of participants in online cancer screening training programs in India
Source: BMC Med Educ. 2020 Jul 13;20:220. doi: 10.1186/s12909-020-02144-y (PMC7359595; doi:10.1186/s12909-020-02144-y)
Supplement: Supplementary file 2 — Additional file 2. Supplementary document 2: Questionnaire for quantitative study. [file 12909_2020_2144_MOESM2_ESM.docx]

**1)What is the main reason for you to quit attending the NICPR-ECHO's Online Cancer Screening Training Program for Medical Officers (CSTP-MO)**

a) Busy at work

b) Not interested

c) I did not have a case to present

d) This course was not relevant to the work I do at my health facility

e) Unable to understand the subject matter

f) No time

**2) What kind of changes in the program do you think will make you attend/ adhere to the program and complete the course??**

a) Change of time

b) Change in the manner the didactic is delivered

c) Change in the format of the course

d) Less number of sessions

e) Module-wise courses for each type of cancers: cervical, breast and oral

f) Change in the presentation format of the subject matter

**3) Please identify your district and state.**
